# Liquid Biopsy in Low-Grade Glioma: A Systematic Review and a Proposal for a Clinical Utility Score

**DOI:** 10.1007/s10571-023-01406-9

**Published:** 2023-09-13

**Authors:** Luca Zanin, Alexandra Sachkova, Pier Paolo Panciani, Veit Rohde, Marco Maria Fontanella, Bawarjan Schatlo

**Affiliations:** 1 Unit of Neurosurgery, Department of Medical and Surgical Specialties, Radiological Sciences and Public Health, University of Brescia, Piazzale Spedali Civili 1, 25123 Brescia, Italy; 2 Institute of Clinical Pharmacology, University Medical Center Göttingen, Georg-August University, 37075 Göttingen, Germany; 3 Department of Neurosurgery, University Medical Center Göttingen, Göttingen, Germany

**Keywords:** Liquid biopsy, Low-grade glioma, Score, Clinical, Systematic, Review

## Abstract

Liquid biopsy research on Low-Grade gliomas (LGG) has remained less conspicuous than that on other malignant brain tumors. Reliable serum markers would be precious for diagnosis, follow- up and treatment. We propose a clinical utility score (CUS) for biomarkers in LGG that mirrors their clinical usefulness. We conducted a PRISMA review. We examined each biomarker classifying them by CUS and Level of Evidence (LOE). We identified four classes of biomarkers: (1). Circulating protein—(a) vitronectin discriminates LGG from HGG (Sn:98%, Sp:91%, CUS: 3, LOE: III), (b) CTLA-4 discriminates LGG from HGG, (cutoff: 220.43 pg/ml, Sn: 82%, Sp: 78%, CUS:3, LOE:III), (c) pre-operative TGF b1 predict astrocytoma (cutoff: 2.52 ng/ml, Sn: 94.9%, Sp: 100%, CUS:3, LOE:VI). (2). micro-RNA (miR)—(a) miR-16 discriminates between WHO IV and WHO II and III groups (AUC = 0.98, CUS:3, LOE: III), (b) miR-454-3p is higher in HGG than in LGG (p = 0.013, CUS:3, LOE: III), (c) miR-210 expression is related to WHO grades (Sn 83.2%, Sp 94.3%, CUS: 3, LOE: III). (3). Circulating DNA—(a) IDH1R132H mutation detected in plasma by combined COLD and digital PCR (Sn: 60%, Sp: 100%, CUS: 3, LOE: III). 4. Exosomes—(a) SDC1 serum levels could discriminate GBM from LGG (Sn: 71%, Sp: 91%, CUS: 2C, LOE: VI). Our investigation showed that miRs appear to have the highest clinical utility. The LOE of the studies assessed is generally low. A combined approach between different biomarkers and traditional diagnostics may be considered.

**Graphical Abstract:**

We identified four main classes of biomarkers produced by LGG. We examined each biomarker, classifying them by clinical utility score (CUS) and level of evidence (LOE). Micro-RNA (miRs) appears to have the highest CUS and LOE.

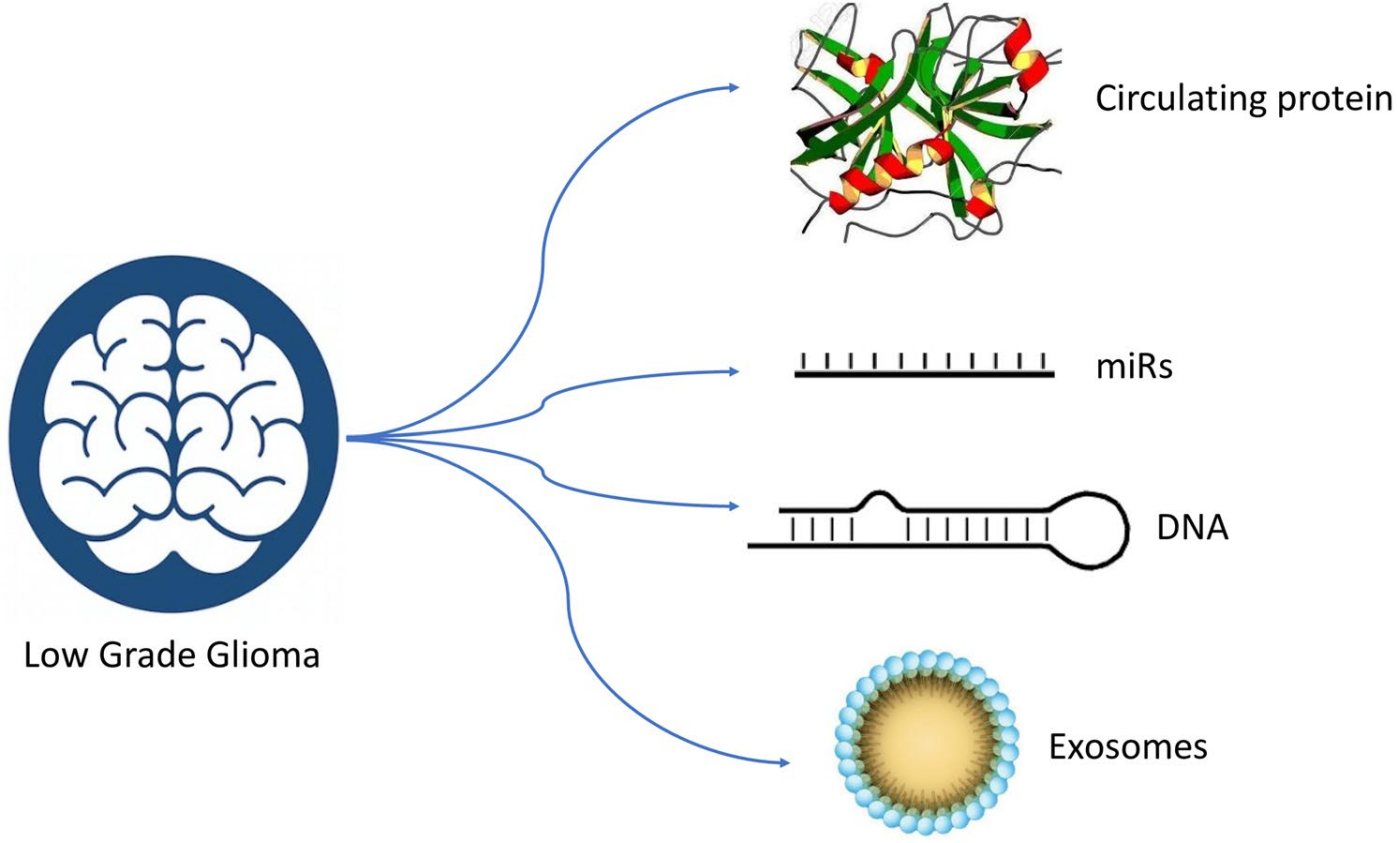

## Introduction

Glioma is the most common primary brain tumor, making up 25.5% of all central nervous system (CNS) malignancies, with an incidence of 25,000 new cases per year in the United States and pooled global incidence of 3.38 per 100,000 person-year(Ostrom et al. 2017). Intense research has begun recently regarding liquid biopsy in high-grade glioma (HGG), identifying several molecules to diagnose and monitor these tumors over time. Scientists have made international revision efforts to take stock of liquid biopsy techniques in neuro-oncology. Conversely, they paid less attention to low-grade glioma (LGG), slower-growing tumors, which may require a longer, more sensitive, and less invasive follow-up. Different patient series have proven that OS (Overall Survival) at 5 and 10 years in the astrocytoma group were respectively about 90 and 50%(Claus et al. 2015).

### Tumor Markers

Tumor markers exist for various types of malignancies (e.g., colon cancer, prostate cancer). Well-conducted randomized controlled trials of numerous kinds of cancer validated their usefulness. For colon cancer, preoperative carcinoembryonic antigen (CEA) has prognostic significance (Wolmark et al. 1984). CA 19–9 levels are significantly associated with survival in patients with inoperable pancreatic cancer (Maisey et al. 2005). Similarly, pituitary hormones (e.g., TSH, ACTH, prolactin) are helpful in the diagnosis and follow-up of patients with pituitary adenomas and prolactinomas (Wass et al. 2011).

### Biomarkers in Biological Fluids

The discovery of a reliable serum marker would be a valuable tool for non-invasive diagnosis, follow- up and guide treatment for LGG patients. Despite regular publications on novel biomarkers for glioma, none is currently in everyday use. Such a marker may facilitate disease management by providing a differential diagnosis, guiding treatment, and predicting recurrence. The present article provides a review of the available literature and aims to discuss the most relevant studies on LGG biomarkers. Moreover, applying the tumor marker utility grading system (TMUGS) (Hayes et al. 1996), we propose a clinical utility score (CUS) for liquid biopsy techniques that mirror the utility of a specific biomarker in the clinical routine.

## Materials and Methods

### Search Strategy

We searched in PubMed, MedLine, Cochrane Library and Google Scholar databases for articles published between January 1, 1960, and October 31, 2022, with the terms “low-grade glioma,” “low-grade brain tumor,” “liquid biopsy,” “CSF marker,” “blood marker,” “miRNA,” “mRNA” used in “AND” and “OR” combinations. We conducted the systematic review according to the PRISMA guidelines (Moher et al. 2015).

### Inclusion and Exclusion Criteria

Inclusion criteria were all the following: (1) studies written in the English language, (2) studies dealing with LGG, (3) studies reporting analysis of Cerebrospinal fluid (CSF), blood, or urine biomarkers, (4) studies reporting sensibility and specificity value for the biomarker analyzed, or receiver operating characteristic (ROC) curve analysis. Exclusion criteria were as follows: (1) studies reporting biomarkers obtained by invasive methods (e.g., tumor excision, biopsy), (2) studies reporting non-glial tumors (3), studies focused exclusively on pediatric tumors.

### Quality Assessment and Data Extraction

We imported the articles into the reference management software Zotero (version 5.0.96.2) and removed the duplicates. We used Rayyan software (Ouzzani et al. 2016) to perform a systematic literature review. A.S. and L.Z. independently examined titles and abstracts of retrieved records and excluded non-relevant citations. A.S. and L.Z. double-checked full-text papers and resolved disagreements through a discussion with a third reviewer: B.S.

We included eligible articles in a database listing each biomarker's biological, radiological, histological, and clinical variables.

Markers were categorized according to their use, i.e., screening, differential diagnosis, follow up, and prognosis.

Applying the tumor marker utility grading system (TMUGS) on LGG (Hayes et al. 1996), we proposed the CUS shown in Table 1. Two independent reviewers, P.P.P and L.Z., graded each article for its CUS and Level Of Evidence (LOE) (Burns et al. 2011). A debate with a third reviewer, M.F., agreed upon any divergences. Table 2 shows the LOE description.

**Table 1:** The table shows the description of our proposed clinical utility score (CUS) for tumor biomarkers, adopting Hayes’ tumor marker utility grading system (TMUGS) (Hayes et al. 1996)

Utility grade	Description
0	Marker evaluated for a specific use: data demonstrate it has no utility. The marker should not be ordered for that clinical use
NA	Data are not available for the marker for that use because marker has not been studied for that clinical use
1	Data are suggestive that the marker may correlate with biologic process and/or biologic end point, and preliminary data suggest that use of the marker may contribute to favorable clinical outcome, but no proven conclusions were reached. The marker is still considered highly investigational and should not be used for standard clinical practice
2	Sufficient data are available to demonstrate that the marker correlates with the biologic process and/or biologic end point related to the use and that the marker results might affect favorable clinical outcome for that use. However, the marker is still considered investigational and should not be used for standard clinical practice, for one of three reasons:
	a. The marker correlates with another marker or test that has been established to have clinical utility, but the new marker has not been shown to clearly provide any advantage
	b. The marker may contribute independent information, but it is unclear whether that information provides clinical utility because treatment options have not been shown to change outcome
	c. Preliminary data for the marker are quite encouraging, but the level of evidence (LOE) is too low
3	Marker supplies information not otherwise available from other measures that is helpful to the clinician in decision-making for that use, but the marker cannot be used as sole criterion for decision-making. Thus, marker has clinical utility for that use., and it should be considered standard practice in selected situations
4	Marker can be used as the sole criterion for clinical decision-making in that use. Thus, marker has clinical utility for that use, and it should be considered standard practice

**Table 2:** The table shows the LOE from highest to lowest grade [6]

Level Of evidence (LOE)	Description
Level I	Evidence from a systematic review or meta-analysis of all relevant RCTs (randomized controlled trial) or evidence-based clinical practice guidelines based on systematic reviews of RCTs or three or more RCTs of good quality that have similar results
Level II	Evidence obtained from at least one well-designed RCT (large multi-site RCT)
Level III	Evidence obtained from well-designed controlled trials without randomization (quasi-experimental)
Level IV	Evidence from well-designed case–control or cohort studies
Level V	Evidence from systematic reviews of descriptive and qualitative studies (meta-synthesis)
Level VI	Evidence from a single descriptive or qualitative study
Level VII	Evidence from the opinion of authorities and/or reports of expert committees

## Results

**Fig. 1:**
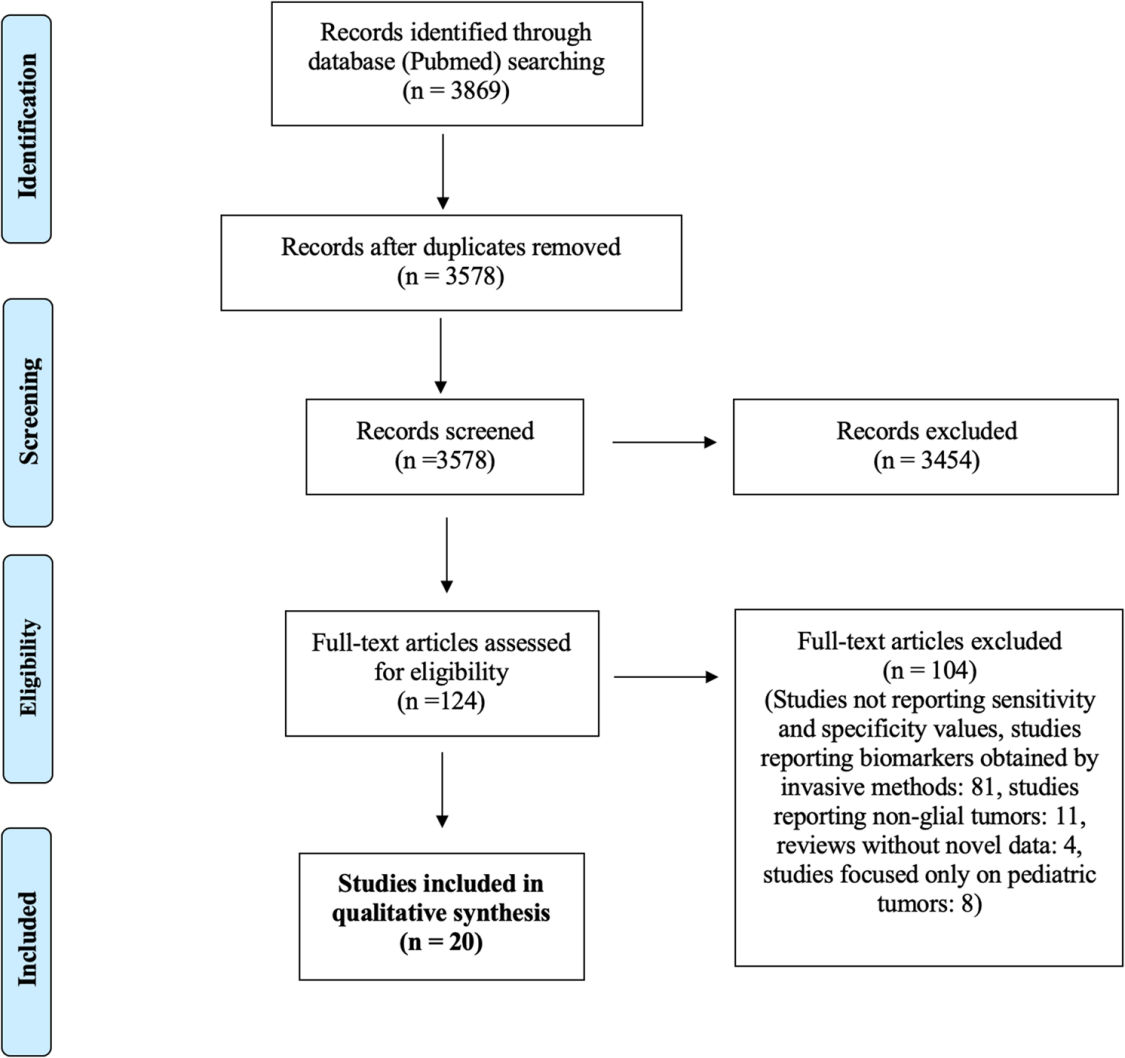
It shows the PRISMA diagram applied to select studies of interest through a systematic literature review process, from identification in the search database to inclusion of data in the review

We identified 3869 papers using the reported keywords. After removing duplicates, we examined all 3578 abstracts and obtained 124 full-text eligible articles: 1689 of the 3578 studies were eliminated because they did not meet points 3 or 4 of the inclusion criteria, 1102 studies were eliminated because they did not meet points 1 and 2 of the exclusion criteria. Seven hundred eighty-seven were eliminated because they did not meet point 3 of the exclusion criteria. We excluded additional 104 studies as they did not meet the inclusion criteria. The final qualitative analysis was conducted on 20 studies with 601 LGG patients. Figure 1 shows the paper selection according to PRISMA. Most of the studies focused on four classes of biomarkers, analyzed on blood, only one study performed an analysis also on CSF: 1. Circulating protein, 2. Circulating MicroRNAs (miRs), 3. Circulating DNA, 4. Circulating cells and exosomes. Table 3 describes the number of studies in our analysis for each biomarker class. We decided to report only the studies able to provide the biomarker's sensitivity and specificity value, as this is essential for application in clinical practice.

**Table 3:** The table shows the number of studies for each biomarker class considered, depending on the type of substance used for the analysis (blood, CSF, or urine)

Biomarker		N° of studies
Circulating protein	Blood	7
	CSF	0
	Urine	1
Circulating miRNA	Blood	5
	CSF	1
Circulating DNA	Blood	2
	CSF	0
Circulating cells and exosomes	Blood	5
	CSF	0

### Circulating Protein

Concerning circulating proteins, we identified seven studies reporting sensitivity and specificity values, as shown in Table 4. Most of these studies were carried out on blood, except for Lombardi who also included urine (Lombardi G et al. 2015). Most studies used the ELISA (enzyme-linked immunosorbent assay) test as an analysis technique, although there have been alternative examples, such as quantitative mass spectrometry (Lombardi G et al. 2015). Of the seven examined studies, none considered the same biomarker. Some results have shown that the marker is not useful in differentiating degrees of WHO gliomas, such as l-CaD (Zheng PP et al. 2005). While other studies, like the one performed by Mao-Hua Chen, succeeded in separating the population of LGG from HGG by having/showing a ROC curve value of 0.995. Loh demonstrated that when the plasma Transforming Growth Factor (TGF) β1 level before tumor removal was ≥ 2.52 ng/ml, a patient having astrocytoma was predicted with a sensitivity of 94.9% and specificity of 100%, with an AUC of 0.98 (*p* < 0.001, [CI] 95% 0.94–1.00) (Loh JK et al. 2012). Adachi-Hayama showed the serum anti-filamin C (FLNC) autoantibody level was significantly higher in LGG than in HGG (LGG vs HGG: *p* = 0.0101, LGG vs healthy controls: *p* < 0.0001) (Adachi-Hayama M et al. 2014). Lombardi calculated the 2-hydroxyglutarate plasma/urine ratio (normalized by creatinine) in glioma patients, and a statistically significant difference was found between the patients with wild-type and mutant isocitrate dehydrogenase (IDH) 1 when the same cases were histologically confirmed: 15.6 ± 6.8 versus 22.2 ± 8.7 (*p* < 0.0001). Mao-Hua Chen demonstrated through ROC curve analysis that serum vitronectin levels discriminated LGG significantly from HGG from healthy volunteers and other CNS tumors (AUC 0.995, 0.993, and 0.996, respectively) (Chen et al. 2016). A study focusing on pretreatment serum lactate levels demonstrated that a high specificity is possible in predicting the WHO grade of the glial lesion. The AUC was 0.705 (95% CI, 0.586–0.804; *p* = 0.0055). Pretreatment serum lactate levels (cutoff value, 2.0 mmol/L) were determined to predict HGG (sensitivity = 45%; specificity = 90.7%) (Shih CC et al. 2017). Liu demonstrated that soluble Cytotoxic T-lymphocyte-associated antigen 4 (sCTLA-4) measured in 26 WHO II patients can discriminate among WHO grades and between glioma and control subjects. Further, ROC curve analysis showed that the cutoff value in blood for sCTLA-4 is 112.1 pg/ml, as well as the sensitivity and specificity of 82.0 and 78.0%, respectively, and a cutoff value of 220.43 pg/ml was the best distinguished in patients between LGG group and HGG group with sensitivity 73.1% and specificity 79.2%. Shorter progression-free survival (PFS) with high sCTLA-4 levels (> 189.64 pg/ml) was found, compared to those with low sCTLA-4 levels (≤ 189.64 pg/ml) (Liu J et al. 2021).

**Table 4:** The table shows the studies reporting a sensitivity and specificity value for the biomarker examined in circulating protein. Data regarding LGG data were extrapolated from the studies

Author	Substance	Patients	Biomarker	Sensitivity (%)	Specificity (%)	Control	Suggested clinical application	LOE	CUS
Zheng, 2005	Blood	15 (II)	l-CaD	91	84	30 (healthy)	l-CaD values cannot differentiate among WHO grades	IV	0
Loh, 2012	Blood	14 (II)	TGFb1	94.9	100	13 (healthy)	plasma TGF-β1 level before tumor removal correlates with glioma histology	VI	3
Adachi-Hayama, 2014	Blood	15 (I) 57 (II)	anti-FLNC autoantibody	72.2	80.5	77 (healthy) 18 (III) 41 (IV)	anti-FLNC autoantibody level higher in LGG	VI	2C
Lombardi, 2015	Blood + Urine	17 (II)	2-hydroxyglutarate plasma/urine ratio	63	76	Same cases histologically confirmed	Identify the IDH status when surgery is not possible	IV	3
Chen M.H., 2016	Blood	54 (I + II)	Vibronectin	98	91	98 (healthy)	Vitronectin level discriminates among WHO grades	III	3
Shih, 2017	Blood	54 (I + II)	Serum lactate levels	59.3 (excluding HGG)	95.7 (excluding HGG)	same cases histologically confirmed	Pretreatment serum lactate has high specificity in predicting WHO grades	IV	2B
Liu, 2021	Blood	26 (II)	CTLA-4	82	78	50 (healthy)	sCTLA-4 values separate among WHO grades and between glioma and control subjects	III	3

From our analysis, considering LOE and CUS, the most promising circulating protein appears to be vitronectin (Chen et al. 2016), sCTLA-4 (Liu J et al. 2021), and TGF-β1 (Loh JK et al. 2012). Although the LOE from these studies is low, the clinical utility of these biomarkers can potentially be of a high standard.

### Circulating miRs

Six studies provided their biomarkers’ sensitivity and specificity values regarding the circulating miRs, as shown in Table 5.

**Table 5:** The table shows the studies reporting a sensitivity and specificity value for the biomarker examined in circulating miRs. Data regarding LGG data were extrapolated from the studies

Author	Substance	Patients	Biomarker	Sensitivity (%)	Specificity (%)	Control	Suggested clinical application	LOE	CUS
Zhi, 2015	Blood	50 (II)	miR-20a-5p, miR-106a-5p, and miR-181b-5p	94	92	50 (healthy)	All 3 miRNAs correlate with OS	III	3
D’Urso, 2015	Blood	8 (II)	miR-15b	100	100	30 (other CNS pathology) 36 (PCNSL) 16 (MTS)	miR-15b discriminates glioma patients from controls	III	3
			miR-16	98	98		miR-16 classifies among WHO grades	III	3
Shao, 2015	Blood	8 (I) 15 (II)	miR-454-3p	99.5	82.9	70 (healthy)	High levels of miR-454-3p correlate with poor OS	III	3
Huang, 2017	Blood	10 (I) 20 (II)	miR-376a	81	82	50 (healthy)	All 3 miRNAs expression separate glioma from controls. Decreased expression were related with higher WHO grade and low KPS	III	3
			miR-376b	82	78				
			miR-376c	90	70				
Kopkova, 2019	CSF	14 (II)	miR-21-3p	73	72	21 (NPH)	miR-30e and miR-140 separate LGG patients from controls let-7b, miR-21-3p, and miR-10a stratify GBM, meningioma and brain metastasis from other brain tumor types	IV	3
			miR-30e	76	75				
			miR-140	76	75				
			let-7b	73	72				
			miR-10a	73	72				
Lan, 2020	Blood	12 (I) 20 (II)	miR-210	83.2	94.3	50 (healthy)	High levels of miR‑210 correlates to poor OS	III	3

Studies conducted on miRs are generally those with larger patient cohorts, with higher evidence levels and, therefore, able to provide the most reliable results. Most studies took place on blood, while only one reported results from CSF (Kopkova A et al. 2019). The technique of quantitative reverse transcription PCR (polymerase chain reaction) was used for all the analyses. Lan demonstrated with 32 LGG patients in the leading case-cohort that high levels of miR-210 correlate to poor OS. MiR-210 levels can distinguish patients with glioma from healthy controls, with an AUC value of 0.856 (95% CI, 0.795–0.917), sensitivity of 83.2%, specificity of 94.3%, positive predictive value of 93.5% and negative predictive value of 70.3%. Furthermore, MiR-210 expression is related to WHO grades, which implies that miR-210 may be a significant predictor of poor prognosis in HGG compared with LGG (Lan et al. 2020).

Three different miRs (miR-20a-5p, miR-106a-5p, and miR-181b-5p) studied by Zhi on 50 patients with LGG (WHO II) were shown to correlate with OS. The diagnostic value of this miR profiling system was assessed by a risk score function (RSF). The authors used a linear combination of the expression level for each miR in order to calculate the contribution of each miR to the RSF. ROC curves were then used to evaluate the diagnostic effects of the profiling and to find the appropriate cutoff point, identified as RSF = 5.649. The authors used Kaplan–Meier survival analysis to compare the participants with high-risk and low-risk scores. The participants with high-risk scores had a poorer survival rate than those with low-risk scores (*p* = 0.002) (Zhi F et al. 2015).

D'Urso demonstrated that miR-15b could provide evident separation between the glioma patients group and those without tumors. With plasma relative expression levels for miR-15b higher than 4, they could select all 30 glioma patients (sensibility and specificity = 100%). Furthermore, by ROC curves analysis of miR-16, an evident separation was detected between the WHO IV and WHO II and III groups (AUC = 0.98). The authors set the cutoff levels for miR-16 at 0.33 with 98% sensitivity and 98% specificity. The only false-negative results were observed for two patients with anaplastic astrocytoma diagnosis (WHO grade III) (Ivo D’Urso P et al. 2015).

WHO grade was linked to another biomarker as proved by Shao: the plasma levels of miR-454-3p were significantly higher in HGG (WHO grade III and IV) than in LGG (WHO grade I and II) (*p* = 0.013)(Shao N et al. 2015). This result was reinforced by the OS analysis proving a poorer marginally significant survival rate in glioma patients who expressed high levels of miR-454-3p (*p* = 0.040). Furthermore, the ROC curve analysis showed that 1200 fM/L was the optimal cutoff reflecting the ability of the plasma levels of miR-454-3p to differentiate the glioma patients from the controls with 99.05% sensitivity and an 82.86% specificity (AUC 0.9063) [95% (CI): 0.8487–0.9639)] (Shao N et al. 2015).

On the other hand, Huang clearly showed that decreased expression of miR-376a, miR-376b, and miR-376c in patients' serum were related to higher WHO grade (*p* < 0.01) and low KPS (*p* < 0.05). Kaplan–Meier analyses showed that low miR-376a, miR-376b, and miR-376c expression were all independent factors predicting the poor outcome of glioma patients. Nevertheless, serum miR-376a, miR-376b, and miR-376c levels had more significant prognostic values in patients with HGG than those with LGG (Huang Q et al. 2017).

Kopkova calculated CSF levels of let-7b, miR-21-3p, and miR-10a, and they were able to stratify GBM (sensitivity 73% and specificity 75%), meningioma (sensitivity 73% and specificity 72%), and brain metastasis (sensitivity 75% and specificity 71%) from other brain tumor types. Furthermore, let-7c, miR-140, and miR-196a show significantly different levels in HGG and LGG patients' CSF (Kopkova A et al. 2019).

From our analysis, considering LOE and CUS, the most promising miR appears to be miR-16 (Ivo D’Urso P et al. 2015), miR-454-3p (Shao N et al. 2015), and miR-210 (Lan et al. 2020) for the highest levels of sensitivity and specificity achieved in the respective studies; however, all biomarkers in this category appear to be extremely promising.

### Circulating DNA

We identified only two studies that presented sensitivity and specificity values for circulating DNA, as shown in Table 6. Both studies used blood as a substance of analysis.

**Table 6:** The table shows the studies reporting a sensitivity and specificity value for the biomarker examined in circulating DNA. Data regarding LGG were extrapolated from the studies

Author	Substance	Patients	Biomarker	Sensitivity (%)	Specificity (%)	Control	Suggested clinical application	LOE	CUS
Boisselier, 2012	Blood	28 (II)	IDH1 (R132H)	60	100	31 (healthy)	COLD PCR and digital PCR were used to detected in the plasma DNA of 15 out of 25 patients (60%) tumor and in none of the 14 patients harboring an IDH1wt tumor	III	3
Chen, 2013	Blood	15 (I) 17 (II)	Alu element methylation	69	97	30 (healthy)	Alu methylation level correlates with LGG histology	IV	2C

Boisselier focused on identifying the IDH1R132H mutation in plasma samples (80 patients and 31 healthy controls) containing small-size DNA ranging from 150 base pairs (bp) to 250 bp, reaching a specificity of 100%. They combined co-amplification at lower denaturation temperature (COLD) PCR, which preferentially amplifies mutant DNA and digital PCR, which is a highly sensitive approach, to detect the IDH1R132H mutation. It was detected in the plasma DNA of 15 of 25 patients (60%) harboring an IDH1R132H mutated tumor and in none of the 14 patients carrying an IDH1^wt^ tumor. The presence of the IDH1R132H mutation in plasma confirms the diagnosis in patients for which it is impossible to perform a surgical biopsy (Boisselier B et al. 2012).

Chen showed that the median Alu methylation level in patients was 47.30% (interquartile range: 35.40–54.25%), whereas it was 57.90% (interquartile range: 55.25– 61.45%) in the controls. Alu methylation in serum cell-free DNA was significantly lower in glioma patients than in healthy controls (*p* < 0.01), and they found a significant correlation between LGG and HGG both in tumor (*p* < 0.01) and serum (*p* < 0.01) samples. In the serum group, the values were 51.55% (interquartile range, 45.02–55.02%) and 36.50% (interquartile content, 34.05–49.10%), respectively, with statistically significant differences in both sample sets (*p* < 0.01) (Chen J et al. 2013).

From our analysis, considering the LOE and the CUS, the most promising biomarker among circulating DNA seems to be IDH1R132H mutation proposed by Boisselier (Boisselier B et al. 2012).

### Circulating Cells and Exosomes

Our analysis identified five studies involving circulating cells and exosomes concerning LGG, as shown in Table 7. Bao studied Neutrophil Lymphocyte Ratio (NLR) in LGG diagnosis and follow-up. High NLR was associated with a higher tumor grade (*p* < 0.001). Kaplan–Meier survival analyses showed that the high NLR group experienced inferior median OS compared with the low NLR groups (11 vs 32 months; *p* < 0.001) (Bao Y et al. 2018). Gao, examining the polyploidy of chromosome 8 in circulating tumor cells, was unable to demonstrate a statistically significant difference between the WHO grades of the gliomas reviewed (Gao F et al. 2016).

**Table 7:** the table shows the studies reporting a sensitivity and specificity value for the biomarker examined in circulating cells and exosomes

Author	Substance	Patients	Biomarker	Sensitivity (%)	Specificity (%)	Control	Suggested clinical application	LOE	CUS
Gao, 2016	Blood	5 (I + II)	CTCs containing polyploidy chromosome 8	77	NA	10 (healthy)	No statistical difference of CTC incidence and count was observed in different pathological subtypes or WHO grades of glioma	IV	1
Liang, 2018	Blood	53 (II)	SII	75	66	same cases histologically confirmed	The value of 392.48/L was selected as the most optimal cutoff value for SII for discriminate HGG from LGG	IV	2B
Bao, 2018	Blood	57 (I + II)	NLR	80.5	44.3	same cases histologically confirmed	High NLR was associated with a higher tumor grade (P = 0.001), low NLR was associated with LGG	IV	2B
Santangelo, 2018	Blood	2 (I) 13 (II)	miR-21 miR-124-3p miR-222	75	47	30 (healthy)	miR-21 levels separate patients with HGG from those with LGG (AUC 0.83)	IV	2A
Chandran, 2019	Blood	17 (II)	SDC1	71	91	same cases histologically confirmed	SDC1 levels can discriminate GBM from LGG	VI	2C

Chandran proved that Syndecan-1 (SDC-1) serum levels as plasma extracellular vesicle (plEV) constituent could discriminate GBM from LGG. ELISA-based quantification of plEV^SDC1^ could discriminate between GBM and LGG patients with a higher significance level (*p* < 0.0002) (Indira Chandran V et al. 2019).

Another study on inflammation indices was conducted by Liang. They proposed the systemic immune-inflammation index (SII), a new inflammatory marker based on platelets, neutrophils, and lymphocytes in the complete blood count. They identified the value of 392.48/L as the most optimal cutoff value for discriminating HGG from LGG (AUC = 0.773) (Liang R et al. 2018).

Santangelo conducted a study on three miRs extracted from plEV, and miR-21 levels separate patients with HGG from those with LGG (AUC 0.83) (Santangelo A et al. 2018).

Considering the LOE and the CUS, our analysis shows that the most promising biomarker among circulating cells and exosomes is SDC-1 (Indira Chandran V et al. 2019). In general, the LOE of this biomarker class are very low, and the CUS does not reach satisfactory grades.

We ranked each paper in this review based on LOE and clinical utility grade. The result is shown in Table 8. From our analysis, there are no studies with LOE I and II. In general, as the level of evidence increases, so does the level of clinical utility, with some exceptions (Zheng PP et al. 2005).

**Table 8:** the table shows the studies examined classified by LOE and CUS

		Clinical utility score							
Level of Evidence		**0**	**NA**	**1**	**2A**	**2B**	**2C**	**3**	**4**
	I	0	0	0	0	0	0	0	0
	II	0	0	0	0	0	0	0	0
	III	0	0	0	0	0	anti-FLNC autoantibody;	IDH1^(R132H)^; miR-20a-5p, miR-106a-5p, and miR-181b-5p; miR-15b, miR-21; miR-454-3p; vibronectin; miR-376a, miR-376b, miR-376c; miR-210; CTLA-4;	0
	IV	l-CaD;	0	0	miR-21, miR-222, miR-124-3p;	SII; serum lactate levels; NLR, PLR, MLR, RDW;	Alu element methylation;	2-hydroxyglutarate plasma/urine ratio; miR-30e, miR-140, let-7b, mR-10a;	0
	V	0	0	0	0	0	0	0	0
	VI	0	0	0	0	0	Syndecan-1;	TGFb1	0
	VII	0	0	0	0	0	0	0	0

The bold values (0 - NA - 1 - 2A - 2B - 2C - 3 - 4) refer to the Clinical utility score

I - II - III - IV - V - VI - VII are the Levels of Evidence

## Discussion

Lately, there has been an increasing interest in liquid biopsy techniques for managing brain tumors, both primary and secondary ones (Boire et al. 2019; Soffietti et al. 2022). We believe that to make a difference compared to traditional diagnostic tools in the clinical practice of LGG, the liquid biopsy techniques must solve at least 1 of these four main issues:

1. Detecting tumor histology;
2. Detecting a growing recurrence;
3. Detecting tumor evolution to a higher level of malignancy;
4. Detecting tumor response to the therapy in progress.

All these issues may be carried out with an accuracy that should not be inferior to the traditional invasive diagnostics.

### Diagnostic Testing Accuracy

We decided to exclude all the studies that did not report a sensitivity and specificity value of the analyzed biomarker because diagnostic tests in patient care must be evidence-based (Bartol 2015; Shreffler and Huecker 2022). Many studies showed exciting results, but the absence of the sensitivity and specificity parameters of the examined biomarker prevented us from considering them functional in the everyday clinical context (Zhang L et al. 2014; Lai et al. 2015; Chen et al. 2016; Tabibkhooei A et al. 2020; Piazza et al. 2022).

#### CUS

We proposed a CUS that reflects the utility of a specific biomarker in the clinical routine.

Non-invasive liquid biopsy techniques may be useful in many cases, e.g., whereas it is not safe to perform surgery due to the anatomical position of the tumor or the patient's general condition. Liquid biopsy can play a significant role even with traditional diagnostic techniques, like MRI. In a radiologically doubtful LGG with areas of color change, the possibility of coupling the serum or CSF dosage of biomarkers such as miR16, miR30e, miR140, which are associated with a malignant evolution of the disease allows strengthening the diagnosis significantly (Ivo D’Urso P et al. 2015).

If we associate the use of these biomarkers in a standardized way alongside traditional radiology, however, the opposite situation would also be possible: even with an MRI undeniably showing an LGG, in the presence of some very sensitive biomarkers, such as miR-376c, TGF-b1, or vitronectin, a progression may by suspected (Loh JK et al. 2012; Lan F et al. 2020). This could represent an avenue toward personalized care and targeted therapy.

Liquid biopsy techniques should be used in the context of integration with the diagnostics presently existent for diagnosing and following cerebral gliomas (MRI and surgical histological biopsy). A thought-provoking feature is that liquid biopsy can obtain cellular information produced by the entire tumor microenvironment and not only from the biopsied part (Corcoran and Chabner 2018).

None of the biomarkers reached level 4, which corresponds to the power of that biomarker to satisfy the specific clinical demand as the sole criterion for clinical decision-making in that use. For this reason, while we wait for more studies with higher LOE, we speculate it is prudent to integrate the routinely used diagnostic tools with these new techniques only if they have achieved a CUS of at least 3 and a LOE of at least III.

### Level of Evidence

The central problem addressed in this systematic review of the literature concerned LOEs, which were generally low. The few meta-analyzes in literature are provided only on limited classes of biomarkers, analyzed with the same laboratory investigation (He et al. 2020).

As regards the LGG, we did not find any meta-analyzes or dedicated systematic reviews. Likewise, the wide range of techniques impedes our efforts of constructing meta-analyzes on the available data.

### Cost-Effectiveness Issues

Although few papers have addressed the problem, one of the issues raised about liquid biopsy techniques is the economic load that these procedures introduced in treating pathologies already burdened by high costs for the healthcare system. However, several studies conducted on liquid biopsy applications in different clinical areas have shown how the cost–benefit ratio is sustainable (Ezeife et al. 2022; Englmeier et al. 2022).

### On-Going Trials

The number of clinical trials currently underway investigating biomarkers with liquid biopsy techniques is relatively low (five trials in phase 1, two trials in phase 2, and four observational trials). Most of these, however, are focused on GBMs and high-grade glial lesions (Soffietti et al. 2022).

### Combined Approach

Although we do not have reliable data about a combined approach, we might consider a merged use of different biomarkers with higher CUS and LOE, e.g., COLD PCR was used to detect IDH1R132H mutation in blood with 100% specificity but with low sensitivity (Boisselier B et al. 2012), while a high level of miR-454-3p in blood showed a 99.5% sensibility for poor OS (Shao N et al. 2015). Therefore, in case of both absence of IDH1R132H mutation and high level of miR-454-3p we could identify a glioma wt and predict a poor prognosis.

We believe that once the most clinically valuable biomarkers have been identified, combined studies could analyze the same group of patients if a merged use increases their diagnostic power.

## Limitations

According to our findings miRs possess the utmost clinical utility; nevertheless, numerous studies encompassed within this review concerning miRs exhibit a considerably small sample size. Such small sample size is grossly inadequate for constructing an accurate and resilient diagnostic model for any biomarker. Hence, it is imperative to underscore the shortcomings present in the existing literature and assert that future investigations must be conducted with a substantial cohort of patients before any definitive conclusions can be drawn.

## Conclusion

Our investigation showed that miR studies appear to have the highest clinical utility. The LOE of the evaluated studies is generally low. Many biomarkers with different procedures and multiple goals were tested, and this prevents a meta-analytical analysis of the results. Certainly, it appears mandatory to develop prospective randomized trials with standardized protocols and procedures focused on the molecules that have given the best results in the retrospective and case–control studies conducted so far. Nevertheless, we could speculate that a combined approach of the different techniques examined in this review could already lead to significant and personalized progress in the LGG patients’ management.

## Data Availability

Not applicable.

## Abbreviations

AGR: Albumin/globulin ratio
B7-H3: B7 homolog 3 protein
BCL2L1: Bcl-2-like protein-1
CEA: Carcinoembryonic antigen
CCDC28A: Coiled-coil domain containing 28A
CNS: Central Nervous System
COLD: Co-amplification at lower denaturation temperature
CUS: Clinical utility Score
CSF: Cerebrospinal fluid
DAPK1: Death-associated protein kinase 1
ELISA: Enzyme-linked immunosorbent assay
exoDNA: Exosomal DNA
FLNC: Filamin C
GBM: Glioblastoma
GFAP: Glial fibrillary acidic protein
GZMB: Granzyme **B**
HGG: High grade glioma
HLA: Human leukocyte antigen
hTERT: Human telomerase reverse transcriptase
IDH: Isocitrate dehydrogenase
IL: **I**nterleukin
IGFBP2: Insulin-like growth factor binding protein-2
IRF-1: Interferon regulatory factor-1
l-CaD: Low-molecular weight caldesmon
LGG: Low-Grade glioma
LMR: Lymphocytes to monocytes
LOE: Level of evidence
NLR: Neutrophyl-to-lymphocyte ratio
MDA: Malondialdehyde
MDSCs: Myeloid-derived suppressor cells
MGMT: Methylguanine-DNA-methyltransferase
miR: MircoRNA
MMP-9: Matrix metallopeptidase-9
OS: Overall survival
PAI-1: Plasminogen activator inhibitor-1
PARP-1: Poly-[ADP-ribose]-polymerase-1
PCR: Polymerase chain reaction
PFS: Progression-free survival
plEV: Plasma extracellular vesicle
PLR: Platelet-lymphocyte ratio
PNI: Prognostic nutrition index
PRISMA: Preferred reporting items for systematic reviews and meta-analyses
RDK: Protein Kinase, DNA-Activated, Catalytic Subunit
ROC: Receiver operating characteristic
RSF: Risk score function
SHP-1: Protein tyrosine phosphatase (PTP) that contains an SH2 domain
SH3GL1: SH3 Domain containing GRB2 Like** 1**
SNPs: Single-nucleotide polymorphism
SII: Systemic immune-inflammation index
SDC-1: Syndecan-1
sCTLA-4: Soluble Cytotoxic T-Lymphocyte Antigen 4
TGF: Transforming Growth Factor
TIMP-3: Metalloproteinase inhibitor 3
TLR-2: Toll-like receptor 2
TMUGS: Tumor marker utility grading system
TP53: Tumor protein P53
TREM: Triggering receptor expressed on myeloid cells
TSP-1, TSP-2: Thrombospondin-1, thrombospondin-2
VEGF-A: Vascular endothelial growth factor A
wt: Wild type
WHO: World Health Organization
